# Surgical resection of recurrent extrahepatic hepatocellular carcinoma with tumor thrombus extending into the right atrium under cardiopulmonary bypass: a case report and review of the literature

**DOI:** 10.1186/s40792-016-0241-7

**Published:** 2016-10-11

**Authors:** Mineto Ohta, Chikashi Nakanishi, Naoki Kawagishi, Yasuyuki Hara, Kai Maida, Toshiaki Kashiwadate, Koji Miyazawa, Satoru Yoshida, Shigehito Miyagi, Yukihiro Hayatsu, Shunsuke Kawamoto, Yasushi Matsuda, Yoshinori Okada, Yoshikatsu Saiki, Noriaki Ohuchi

**Affiliations:** 1 Division of Advanced Surgical Science and Technology, Graduate School of Medicine, Tohoku University, 1-1 Seiryou, Aoba, Sendai, 980-8574 Japan; 2 Division of Cardiovascular Surgery, Graduate School of Medicine, Tohoku University, 1-1 Seiryou, Aoba, Sendai, 980-8574 Japan; 3 Department of Thoracic Surgery, Institute of Development, Aging and Cancer, Graduate School of Medicine, Tohoku University, 1-1 Seiryou, Aoba, Sendai, 980-8574 Japan

**Keywords:** Hepatocellular carcinoma, Recurrence, Right atrial tumor thrombus, Cardiopulmonary bypass

## Abstract

**Background:**

Recurrent hepatocellular carcinoma accompanied by a right atrial tumor thrombus is rare. No standard treatment modality has been established. Surgical treatment may be the only curative treatment; however, surgery has been considered high risk. We herein describe a patient who underwent resection of a recurrent right atrial tumor thrombus under normothermic cardiopulmonary bypass on a beating heart.

**Case presentation:**

A 60-year-old man underwent a right hepatectomy for hepatocellular carcinoma with diaphragm invasion. During the preoperative cardiac screening, he was diagnosed with an old myocardial infarction with triple-vessel coronary disease. Percutaneous coronary intervention was performed for the left anterior descending artery and left circumflex coronary artery. High-grade stenosis remained in his right coronary artery. Nine months later, computed tomography showed recurrent hepatocellular carcinoma in the diaphragm and a tumor thrombus extending from the suprahepatic inferior vena cava into the right atrium. Surgical resection of the recurrent tumor was performed through a right subcostal incision with xiphoid extension and median sternotomy. The recurrent tumor was incised with the diaphragm and pericardium. Intraoperative ultrasonography revealed that the tumor thrombus was free from right atrium wall invasion and that the right atrium could be clamped just proximal to the tumor thrombus. The right atrium, infrahepatic vena cava, left and middle hepatic veins, and hepatoduodenal ligament were encircled. Cardiopulmonary bypass was performed to prevent ischemic heart disease caused by intraoperative hypotension. Total hepatic vascular exclusion was then performed under normothermic cardiopulmonary bypass on heart beating. The inferior vena cava wall was incised. The tumor thrombus with the diaphragmatic recurrent tumor was resected en bloc. The patient had a favorable clinical course without any complications.

**Conclusion:**

The recurrent hepatocellular carcinoma in the diaphragm and the right atrial tumor thrombus were safely resected using normothermic cardiopulmonary bypass on heart beating.

## Background

Hepatocellular carcinoma (HCC) with a right atrial tumor thrombus (RATT) is associated with a poor prognosis because heart failure, pulmonary embolism, or cancer progression results in an unavoidable fatal outcome. Transcatheter arterial chemoembolization, radiotherapy, or surgery is used to treat the tumor thrombus (TT); however, no standard treatment modality has been established [1]. Improvements in surgical procedures such as cardiopulmonary bypass (CPB) or total hepatic vascular exclusion (THVE) have allowed for the performance of aggressive surgery [2]. Surgical resection may be the only curative treatment that increases survival [3, 4]. However, few reports have described HCC resection with a recurrent RATT. We herein present a case involving resection of a recurrent RATT using normothermic CPB on a beating heart.

## Case presentation

A 60**-**year-old man underwent right hepatectomy with the omentum and diaphragm for treatment of HCC with diaphragm invasion. He had chronic hepatitis C with a sustained virologic response to interferon treatment at 37 years old. An old myocardial infarction with triple-vessel disease was found, and percutaneous coronary intervention was performed for the left anterior descending artery and left circumflex artery before surgery. High-grade stenosis in his right coronary artery was followed up without intervention because of the presence of a small area of myocardial perfusion. After right hepatectomy, he had a favorable clinical course without any complications. After discharge from the hospital, he was followed up by α-fetoprotein (AFP) measurement, des-γ-carboxy prothrombin (DCP) measurement, and ultrasonography. However, a recurrent tumor was found by follow-up computed tomography (CT) 9 months after surgery.

**Fig. 1:**
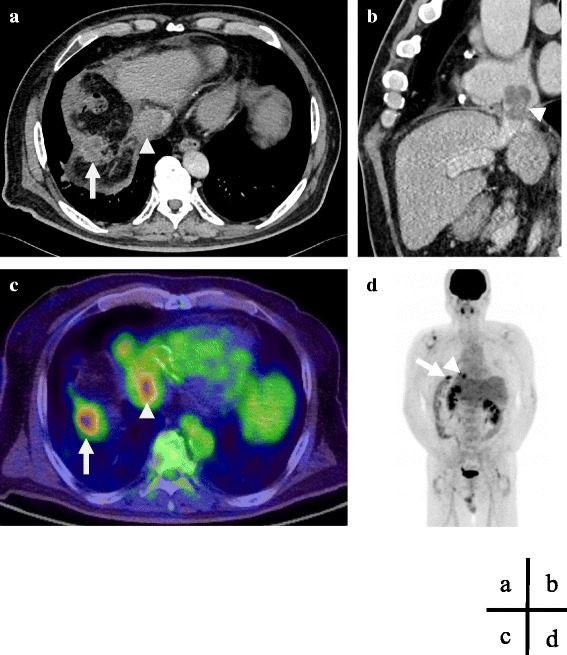
Preoperative imaging studies. **a** Abdominal enhanced computed tomography revealed the tumor in the diaphragm (*arrows*) and the tumor thrombus (*arrowhead*). **b** The tumor thrombus extended from the retrohepatic inferior vena cava to the right atrium. **c** Positron emission tomography revealed the physiologic uptake of 18-fluorodeoxyglucose in both the diaphragm and right atrial tumor, **d** suggesting no remote metastasis

The physical examination of the patient was unremarkable. His laboratory data were within the normal ranges. The serum levels of tumor markers were as follows: carcinoembryonic antigen, 1.9 ng/mL; AFP, 3.6 ng/mL; *lens culinaris* agglutinin A-reactive α-fetoprotein, 33 %; and DCP, 20 mAU/mL. Abdominal CT showed a recurrent tumor in the diaphragm and a TT extending from the inferior vena cava (IVC) to the right atrium (RA) (Fig. 1a, b). Positron emission tomography/CT images depicted the physiologic uptake of fluorine-18 fluorodeoxyglucose in both the diaphragm and RA tumor (Fig. 1c), but no remote metastasis was observed (Fig. 1d). Echocardiography demonstrated that the RATT did not cause tricuspid valve occlusion, and the cardiac function was good. Coronary angiography showed that the left anterior descending artery and left circumflex artery were patent and that the right coronary artery had a high-grade stenosis; the findings regarding his right coronary artery were the same as those observed during the primary operation. A cardiovascular medicine physician assessed that the intervention for the stenosis had little effect on his life prognosis. Because these examinations revealed that the tumor was not invading the RA wall and that the patient had well-preserved heart function, resection was performed.

**Fig. 2:**
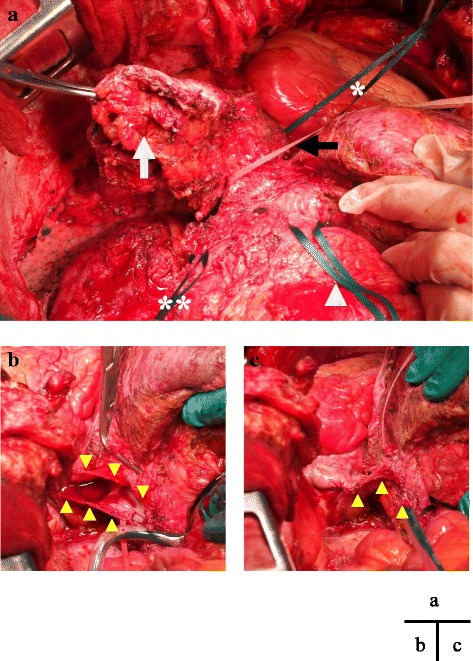
Operative findings. **a** *White arrow* shows the recurrent tumor in the diaphragm. The right atrium, infrahepatic inferior vena cava (IVC), left and middle hepatic veins, and hepatoduodenal ligament were encircled with vascular tape. *Single asterisk* right atrium, *double asterisk* IVC, *white arrowhead* hepatoduodenal ligament, *black arrow* IVC and hepatic vein. **b** The IVC wall was incised (*yellow arrowheads*), and the right atrial tumor thrombus with the diaphragmatic tumor was removed en bloc. **c** The IVC wall was sutured closed in a simple continuous pattern

**Fig. 3:**
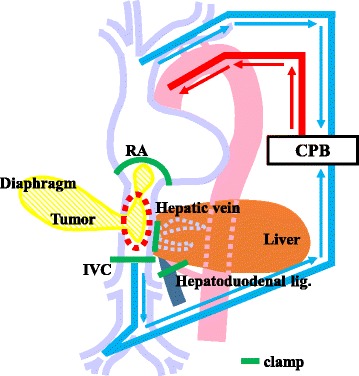
Cardiopulmonary bypass was established with cannulation of the ascending aorta, right femoral vein, and superior vena cava for drainage. The right atrium, infrahepatic inferior vena cava, hepatic vein, and hepatoduodenal ligament were clamped (*green line*). The inferior vena cava wall was incised (*red dotted line*), and the tumor was resected en bloc. *CPB* cardiopulmonary bypass, *RA* right atrium, *IVC* inferior vena cava

Surgery was performed through a right subcostal incision with xiphoid extension and median sternotomy. There was no ascites or peritoneal dissemination. The adhesions involving the left liver, transverse colon, and diaphragm were dissected. The recurrent tumor of the diaphragm was incised with the pericardium. Intraoperative histological examination revealed no tumor in the resected diaphragm stump. Intraoperative ultrasonography showed that the TT was free from RA wall invasion and that the RA could be clamped just proximal to the RATT. The RA, infrahepatic vena cava, left and middle hepatic veins, and hepatoduodenal ligament were encircled with vascular tape (Fig. 2a). We decided to use normothermic CPB to minimize the ischemic damage to the heart caused by intraoperative hypotension. CPB was established with cannulation of the ascending aorta, right femoral vein, and superior vena cava for drainage. Preceding THVE, CPB was started under heparin administration (25,000 units). THVE was performed under normothermic CPB on heart beating as described below (Fig. 3). First, the hepatoduodenal ligament was clamped to stop the inflow to the liver. Next, the RA, infrahepatic vena cava, and left and middle hepatic veins were clamped. The IVC wall was incised (Fig. 2b), and the RATT with the diaphragmatic tumor was removed en bloc. The IVC wall was sutured closed in a simple continuous pattern (Fig. 2c). The THVE and CPB times were 12 and 39 min, respectively. The diaphragm defect was repaired with a Gore-Tex® Soft Tissue Patch (W.L. Gore & Associates Technologies Co., Ltd., Tokyo, Japan). The surgical time was 739 min, and the intraoperative blood loss was 5881 mL.

The patient had a favorable clinical course without any complications and was discharged on postoperative day 24. The diaphragm tumor and TT were pathologically diagnosed as poorly differentiated HCC. The diaphragmatic HCC had invaded the pericardium. Viable tumor cells were present in both the surgical stump of the diaphragm and IVC. The patient began taking sorafenib on day 53 postoperatively. Although magnetic resonance imaging revealed a recurrent lesion in the right thoracic cavity and vertebral body, the patient survived for 10 months after surgery and was still alive at the time of this writing.

### Discussion

The incidence of HCC with a RATT or IVC TT usually ranges from 1 to 4 % [5–7]. Recurrent HCC accompanied by a RATT is rarely encountered. A RATT may result in sudden death because of pulmonary embolism or heart failure. Therefore, this condition should be surgically treated as soon as possible [8–10]. Without any treatment, the survival duration reportedly ranges from 2.4 to 2.7 months [11, 12].

The strategy for treatment of HCC with a RATT usually involves combinations of surgery, radiotherapy, transarterial chemoembolization, and chemotherapy [13]. However, the standard treatment modality for recurrent tumors remains controversial. Surgery can prevent sudden death caused by a RATT, and patients can achieve long-term survival [3, 14]. Even in patients undergoing a noncurative operation for a RATT, surgical resection might be beneficial. After prevention of the life-threatening condition, these patients may have the opportunity to be treated with adjuvant chemotherapy [4].

The problem associated with surgical treatment of a TT is the high operative risk to the patient. Procedures such as THVE, CPB, and hypothermic cardiocirculatory arrest have been used to minimize surgical stress [3]. THVE can decrease operative bleeding while the TT inside the IVC is removed [15]. CPB is used to preserve the intraoperative circulation. Hypothermic cardiocirculatory arrest also has the advantage of reducing operative bleeding and liver damage [16]. Although CPB has potential risks of coagulation dysfunction, transient immunosuppression, and tumor dissemination [17, 18], no differences in clinical outcomes have been reported [19]. In the present case, the patient had an old myocardial infarction and high-grade stenosis remained in the right coronary artery. In the preoperative conference with the cardiovascular surgeon, we discussed how to extract the tumor and prevent the cardinal damage. Preoperatively, it seemed that we would have difficultly extracting the RATT without an atriotomy. Moreover, because of the patient’s coronary disease, we were planning to perform coronary artery bypass grafting (CABG) if we encountered the need to stop the heart from beating to extract the tumor. After the preoperative conference, we prepared for the CPB. In fact, the RATT was free from RA wall invasion and the RA could be clamped just proximal to the RATT. However, when the venous return decreased by hemostasis of the IVC, the patient’s blood pressure dropped. Then, we performed normothermic CPB without stopping the heart to avoid ischemic damage. THVE and CPB helped to reduce bleeding and preserve the operative circulation, respectively. Although the RATT might have been extracted with veno-venous bypass, we used CPB to avoid intraoperative hypotension.

There are many surgical case reports for HCC with RATT [20]; however, surgery for recurrent RATT is rare. Our search of the English literature (PubMed) revealed four cases of resection of a recurrent HCC with a RATT [21–24]. Two of these four cases involved CPB and hypothermic circulatory arrest; both used CPB to reduce intraoperative bleeding caused by atriotomy. Ohwada et al. [23] used CPB because a RATT was extending close to the tricuspid valve. Wu et al. [24] used CPB because it was impossible to perform THVE due to dense adhesions. Conversely, we used CPB in our case to maintain the coronary blood flow. In all cases, the RATT was removed en bloc by incision of the IVC wall without any complications. Although many potential problems are associated with re-resection of a recurrent HCC with a RATT, such as adhesions and liver or heart dysfunction, the combined use of appropriate procedures would minimize the operative morbidity and mortality.

Recurrence of HCC with a RATT is very rare; however, aggressive surgical resection can be performed safely with the appropriate modality. CPB is one such modality that allows for safe resection.

## Conclusions

In summary, we have reported a rare case of HCC recurrence in the diaphragm with a TT extending into the RA. Although the patient had ischemic heart disease, the tumor could be resected without any complications using CPB with heart beating.

## Abbreviations

CPB: Cardiopulmonary bypass
CT: Computed tomography
HCC: Hepatocellular carcinoma
IVC: Inferior vena cava
RA: Right atrium
RATT: Right atrial tumor thrombus
THVE: Total hepatic vascular exclusion
TT: Tumor thrombus
